# Long-term safety of withholding standard prophylaxis in patients with moderate chronic kidney disease

**DOI:** 10.1007/s00330-022-08959-1

**Published:** 2022-06-28

**Authors:** E. C. Nijssen, P. J. Nelemans, R. J. Rennenberg, G. V. van Ommen, J. E. Wildberger

**Affiliations:** Maastricht University Medical Centre, P.O. Box 5800, 6202 AZ Maastricht, the Netherlands

**Keywords:** Contrast media, Acute kidney injury, Chronic kidney disease, Prophylaxis, Clinical practice guidelines

## Abstract

**Abstract:**

In the latest ESUR contrast media guidelines, standard prophylaxis is no longer recommended for patients with moderate chronic kidney disease (CKD). In the absence of solid evidence, guideline updates are often based on indirect evidence and expert opinion. Likewise, evidence supporting the withdrawal of standard prophylaxis in moderate CKD patients was scarce and mostly indirect, but did include one randomised controlled trial evaluating guideline-recommended standard prophylactic intravenous hydration against a group receiving no prophylaxis (A MAastricht Contrast-Induced Nephropathy Guideline (AMACING) trial). Since then, benefits of the updated guideline recommendation for patient and hospital burden have been numerated and were shown to be substantial. The current special report provides data on long-term safety from the AMACING randomised controlled trial.

**Key Points:**

*• In the latest version of ESUR clinical practice guidelines for safe use of contrast media, standard prophylaxis is no longer recommended for patients with moderate chronic kidney disease.*

*• Benefits of this change in recommendations for patient and hospital burden have been numerated. The current report provides data on long-term safety from the AMACING randomised controlled trial.*

*• No disadvantage of withholding prophylaxis could be discerned. Results suggest that, in this population, underlying disease is more relevant for survival and prognosis than contrast administration itself.*

## Introduction

ESUR contrast media guideline recommendations regarding the prevention of post-contrast acute kidney injury (PC-AKI) were last updated in 2018. A major change in recommendations was withholding standard prophylactic intravenous hydration for patients with estimated glomerular filtration rate (GFR) greater than 30 ml/min/1.73 m^2^ [1, 2]. Other international guidelines, including the American College of Radiology guidelines, followed with similar changes [3, 4]. The decision was mainly based on expert opinion, indirect evidence in literature, and one randomised controlled trial (the AMACING trial) [1–5]. This single-centre non-inferiority trial assessed the clinical- and cost-effectiveness of guideline-recommended standard prophylactic intravenous hydration in elective patients with eGFR 30–59 ml/min/1.73 m^2^ combined with risk factors such as old age, diabetes and cardiovascular disease, who were considered to be at high risk of PC-AKI at the time. To date, this remains the only sizeable randomised controlled trial evaluating guideline-recommended standard prophylaxis against a control group receiving no prophylaxis.

Details of methods and primary outcomes of the AMACING trial are published elsewhere [5]. Participants were randomised to receive either standard care prophylactic intravenous hydration (*n* = 328) or no prophylaxis (*n* = 332; see Table 1 for baseline characteristics). All patients included in the trial received intravascular non-ionic iodinated contrast material during an elective intravenous or intra-arterial procedure (contrast-enhanced computed tomography, coronary catheterisation, percutaneous intervention, etc.), and all patients signed informed consent.

**Table 1:** Baseline characteristics of AMACING trial participants

Randomised to	H+ group standard prophylaxis (*n* = 328)	H− group no prophylaxis (*n* = 332)
Men	194 (59%)	213 (64%)
Age at time of contrast administration	71.9 (± 9.3)	72.6 (± 9.3)
BMI (kg/m^2^)	28.6 (± 5.0)	28.7 (± 4.9)
Inpatient	30 (9%)	27 (8%)
Intra-arterial contrast	159 (48%)	160 (48%)
Referral for an interventional procedure	53 (16%)	50 (15%)
Baseline renal function eGFR (ml/min/1.73 m^2^)	47.3 (± 8.0)	47.6 (± 8.0)
Serum creatinine (μmol/L)^1^	118.8 (± 27.6)	117.7 (± 24.6)
Guideline risk groups		
eGFR 45–59 ml/min/1.73 m^2^ and two risk factors	138 (42%)	151 (45%)
eGFR 45–59 ml/min/1.73 m^2^ and diabetes	74 (23%)	65 (20%)
eGFR 30–44 ml/min/1.73 m^2 2^	114 (35%)	115 (35%)
Multiple myeloma or lymphoplasmacytic lymphoma^3^	2 (1%)	1 (0%)
Guideline risk factors		
Diabetes	106 (32%)	109 (33%)
Age > 75 years	140 (43%)	146 (44%)
Prescribed diuretic medication	152 (46%)	155 (47%)
Prescribed non-steroidal anti-inflammatory drug	157 (48%)	162 (49%)
Anaemia^4^	81 (25%)	103 (31%)
Cardiovascular disease	236 (72%)	257 (77%)
Administered volumes (ml)		
300 mg iodine/ml contrast	92 (± 41)	89 ( ± 41)
Intravenous 0.9% sodium chloride		
Pre-hydration	822 (± 486)	0
Post-hydration	809 (± 539)	0
Total	1637 (± 950)	0

Data are *n* (%) or mean (± SD)

*eGFR* estimated glomerular filtration rate

^1^To convert to mg/dl, divide by 88.4

^2^Seventy-six of the 231 patients with eGFR 30–44 ml/min/1.73 m^2^ had diabetes

^3^1 H+ group and 1 H− group multiple myeloma or lymphoplasmacytic lymphoma patient also had an eGFR 30–44 ml/min/1.73 m^2^

^4^Anaemia is defined as a haematocrit value < 0.36 l/l for women and < 0.39 l/l for men

No benefit of prophylactic hydration could be discerned in the prevention of PC-AKI, 1-month and 1-year dialysis or related deaths [5, 6]. Withholding intravenous hydration had advantages in reducing patient and hospital burden, avoiding potentially serious complications such as symptomatic heart failure, and almost halving contrast procedure health-care costs.

Benefits for patient and hospital burden were confirmed 2 years later in a retrospective observational study of Maastricht University Medical Centre before and after data (CINART) [7]. The results showed that the guideline updates led to 89% reduction in complications such as symptomatic heart failure (from 111 to 12 patients per year), 93% reduction in extra hospital admissions for prophylaxis (from 1663 to 119 extra admissions per year) and 91% reduction in costs (from €1.320.696 to €121.992 per year). Now, 5-year results of the AMACING trial are in, providing data on long-term safety.

Below, 5-year post-contrast outcomes are compared between the standard-prophylaxis and no-prophylaxis groups of the AMACING trial. Data was retrospectively obtained from electronic health records. The main 5-year outcome is all-cause mortality, including primary cause distribution. Secondary 5-year outcomes are de novo dialysis, change in eGFR and number of patients with an eGFR declined to below 30 ml/min/1.73 m^2^.

**Fig. 1:**
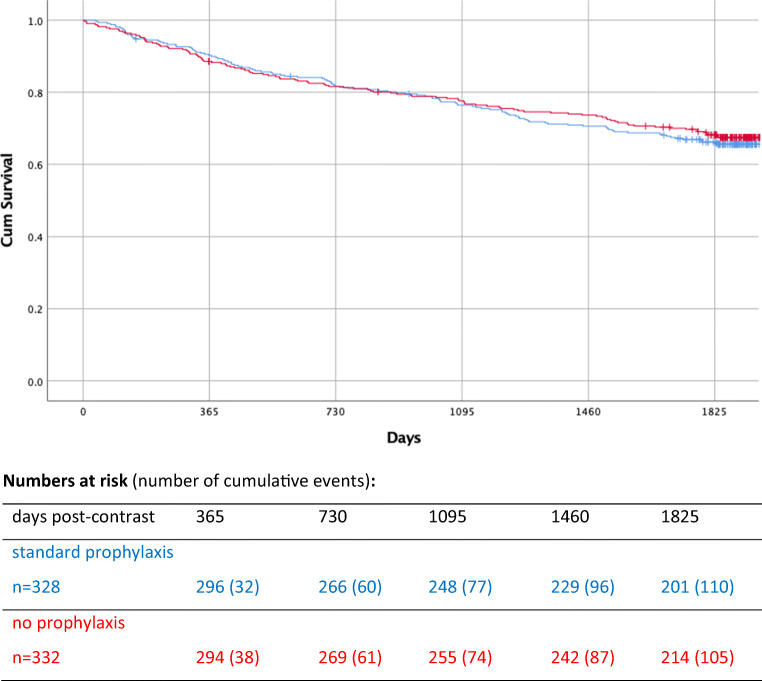
Kaplan-Meier survival curve for the standard prophylaxis (H+) and no prophylaxis (H−) groups of the AMACING randomised controlled trial (RCT). The H+ and H− curves cross over between 2 and 3 years post-contrast. Five-year cumulative probability of survival at the end of the follow-up period is 66.2% for the H+ group and 68.2% for the H− group. Primary causes of death in the standard prophylaxis (H+) and no prophylaxis (H−) groups: cancer 45H+ and 52H−, cardiovascular 27H+ and 26 H−, sepsis 6H+ and 7H−, pneumonia 7H+ and 2H−, respiratory failure 4H+ and 2H−, gastrointestinal 3H+ and 1H−, old age 2H+ and 0H−, cerebral oedema 1H+ and 0H−, heart and renal failure 1H+ and 1H−, multi-organ failure 0H+ and 1H−, unknown 14H+ and 13H−

Long-term follow-up shows that almost one third of the AMACING participants died within 5 years post-contrast (5-year cumulative risk of mortality: 32.8%), but the no-prophylaxis group was not at a disadvantage. Kaplan-Meier survival curves for randomised groups are shown in Fig. 1. The median follow-up period was 1898 days (range 3–2561) and was comparable for both randomised groups (1889 days in the standard-prophylaxis group and 1911 days in the no-prophylaxis group). The two survival curves cross over: the no-prophylaxis group had a slightly higher risk of death during the first 2 to 3 years, and the standard-prophylaxis group had a slightly higher risk of death thereafter. Therefore, the hazard ratio is not constant over time, the proportional hazard assumption does not hold, and it is not possible to describe the difference over time with one hazard ratio and one *p* value. Five-year cumulative probability of survival was slightly higher in the no-prophylaxis group than in the standard-prophylaxis group (68.2% versus 66.2%).

The distribution of known primary causes of death is presented under Fig. 1. Cancer accounted for the highest percentage of deaths in both groups: 40.9% of deaths in the standard-prophylaxis group and 49.5% of deaths in the no-prophylaxis group. The second highest percentage of deaths was caused by cardiovascular events, with similar incidences in both groups (24.5% versus 24.8%). In only three instances did the primary cause include renal function: heart and renal failure was the primary cause of death in one standard prophylaxis patient (< 1% of deaths in that group) and in one no-prophylaxis patient (< 1% of deaths in that group); multi-organ failure was the primary cause of death in one no-prophylaxis patient ( < 1% of deaths in that group).

The higher percentage of cancer deaths in the no-prophylaxis group may account for the crossing over of the survival curves between 2 and 3 years: cancer deaths occurred earlier (median number of days post-contrast 419, interquartile range 192–893) than deaths from other causes (median number of days post-contrast 897, IQR 388–1374; *p* < 0.0001).

**Fig. 2:**
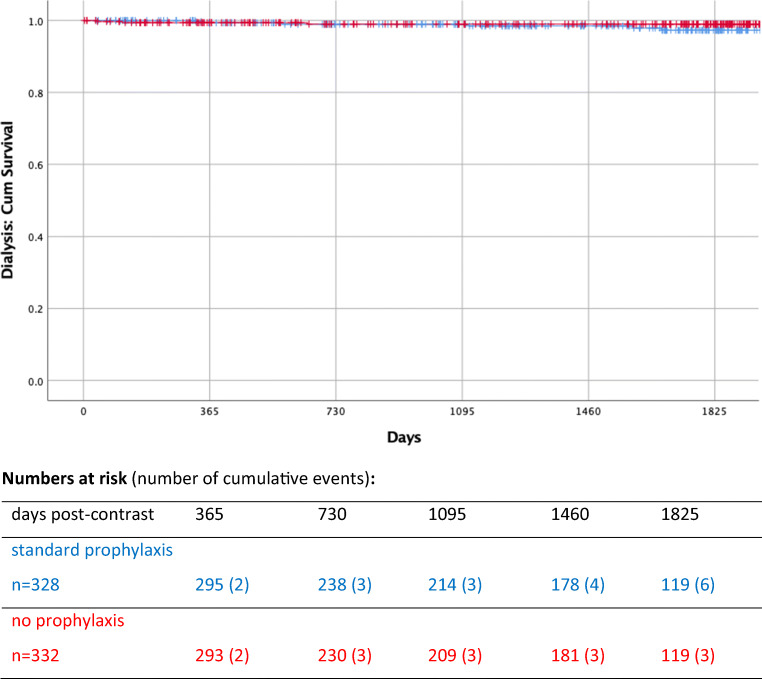
Kaplan-Meier survival curve for dialysis in the standard prophylaxis (H+) and no prophylaxis (H−) groups of the AMACING randomised controlled trial. Curves cross at around 1 year post-contrast. Details of dialysis cases in sequential order (RCT arm, duration of dialysis prior to death, primary cause of death): *37 days post-contrast H−*, 7 days, sepsis; *113 days post-contrast H−*, < 2 days, sepsis; *324 days post-contrast H+*, 66 days, cardiovascular; *350 days post-contrast H+*, < 2 days, sepsis; *562 days post-contrast H+*, 9 days, post-operative cardiogenic shock; *648 days post-contrast H−*, < 1 day, respiratory insufficiency; *1116 days post-contrast H+*, 19 days, cardiovascular; *1588 days post-contrast H+*, 410 days, unknown; *1671 days post-contrast H+*, 116 days, cardiovascular

Kaplan-Meier curves for dialysis are shown in Fig. 2. The median follow-up period for dialysis was 1615 days (range 3–2466) and was comparable for both randomised groups (1599 days in the standard-prophylaxis group and 1635 days in the no-prophylaxis group). Nine cases of de novo dialysis within 5 years post-contrast were recorded: 6 in the standard-prophylaxis group and 3 in the no-prophylaxis group (details of dialysis patients are given under Fig. 2). All 9 patients received dialysis until death. In only three instances was dialysis given over a period greater than 2 months (dialysis duration range 1–410 days). The first two cases of de novo dialysis occurred at 37 and 113 days post-contrast in two no-prophylaxis patients (for a duration of, respectively, 7 and < 2 days prior to death with sepsis as the primary cause in both cases), followed by two cases at 324 and 350 days post-contrast in two standard-prophylaxis patients (for a duration of, respectively, 66 and < 2 days prior to death with cardiovascular event and sepsis as primary causes). The Kaplan-Meier survival curves for dialysis cross at approximately 1 year; therefore, the proportional hazard assumption does not hold and it is not possible to describe the difference over time with one hazard ratio. Nevertheless, there is no indication that withholding prophylaxis confers a higher risk of dialysis in the long term.

**Fig. 3:**
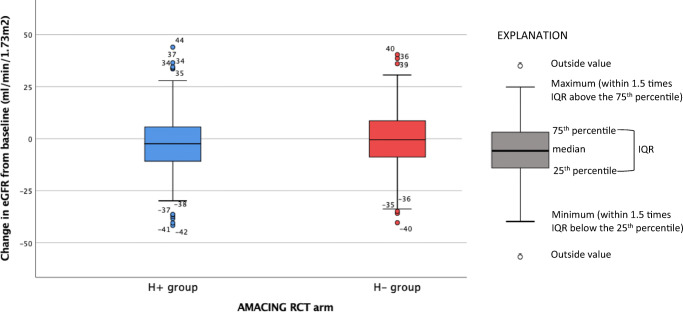
Change in eGFR from baseline at 5 years post-contrast in the standard prophylaxis (H+) and no prophylaxis (H−) groups of the AMACING randomised controlled trial (RCT). eGFR = estimated glomerular filtration rate. Median change in eGFR was − 2.4 ml/min/1.73 m^2^ (IQR − 10.9 to 5.8) for the H+ group and − 0.5 ml/min/1.73 m^2^ for the H− group (IQR − 8.8 to 8.6, *p* = 0.051)

Two hundred one standard-prophylaxis and 214 no-prophylaxis patients were still alive at 5 years post-contrast (Fig. 1). Data on eGFR was available for 353 of these surviving patients (175 standard prophylaxis and 178 no prophylaxis). eGFR was measured at a median of 1898 days post-contrast (range 1477–2466), which was comparable for both groups (standard-prophylaxis patients 1904 days; no-prophylaxis patients 1879 days). Mean eGFR was 44.6 ml/min/1.73 m^2^ (SD 16.6) in the standard-prophylaxis group versus 47.7 ml/min/1.73 m^2^ (SD 16.0) in the no-prophylaxis group (*p* = 0.070). Median change in eGFR was − 2.4 ml/min/1.73 m^2^ (IQR − 10.9 to 5.8) in the standard-prophylaxis group versus − 0.5 ml/min/1.73 m^2^ (IQR − 8.8 to 8.6) in the no-prophylaxis group (*p* = 0.051; see boxplot in Fig. 3). At 5 years, eGFR had fallen to a value below 30 ml/min/1.73 m^2^ in 31/175 (17.7%) of the standard-prophylaxis and 25/178 (14.0%) of the no-prophylaxis patients (*p* = 0.346).

All in all, no disadvantage of withholding prophylaxis could be discerned. These results confirm that the relevant update in the ESUR contrast media guideline is not only beneficial in terms of patient and hospital burden, it is also safe. The high overall mortality, of which only a tiny portion was related to renal function and most was related to cancer and cardiovascular disease, suggests that in this population, despite the elective setting, underlying disease and thus contrast procedure indication is more relevant for survival and prognosis than contrast administration itself.

The next step in this field would be to evaluate the remaining advice on the prevention of PC-AKI for patients with eGFR < 30 ml/min/m^2^, for whom standard prophylaxis is still recommended. However, this is a difficult task due to the relative scarcity of patients with an eGFR < 30 ml/min/m^2^ (elective procedures yield only 0.5% such patients) and their fragility (for example, incidences of 1-month post-contrast mortality and dialysis are approximately 8% and 2%) [8, 9].

## Abbreviations

AMACING: A MAastricht Contrast-Induced Nephropathy Guideline trial
CINART: Contrast-Induced Nephropathy After Reduction of the prophylaxis Threshold
CKD: Chronic kidney disease
eGFR: Estimated glomerular filtration rate
ESUR: European Society of Urogenital Radiology
PC-AKI: Post-Contrast Acute Kidney Injury

## Declarations

### Guarantor

The scientific guarantor of this publication is Prof. J.E. Wildberger.

### Statistics and biometry

One of the authors has significant statistical expertise.No complex statistical methods were necessary for this paper.

### Study subjects or cohorts overlap

Results of the AMACING trial up to 1 month and year have been previously reported in: Nijssen et al, *Lancet* 2017; 389: 1312–22 and Nijssen et al. *EClinicalMedicine by the Lancet* 2018;4–5:109–116. Effects of guideline updates on patient and hospital burden have been previously reported in Nijsen et al. *Eur Radiol* 2020; 30: 4005-4013.

### Methodology

• The AMACING trial is a prospective randomised controlled trial performed at one institution.
